# Spatial variation and mechanisms of leaf water content in grassland plants at the biome scale: evidence from three comparative transects

**DOI:** 10.1038/s41598-021-88678-7

**Published:** 2021-04-29

**Authors:** Ruomeng Wang, Nianpeng He, Shenggong Li, Li Xu, Mingxu Li

**Affiliations:** 1 Key Laboratory of Ecosystem Network Observation and Modeling, Institute of Geographic Sciences and Natural Resources Research, Chinese Academy of Sciences, 11A, Datun Road, Chaoyang District, Beijing, 100101 China; 2 College of Resources and Environment, University of Chinese Academy of Sciences, Beijing, 100049 China; 3 Key Laboratory of Vegetation Ecology, Ministry of Education, Changchun, 130024 China

**Keywords:** Ecology, Evolutionary ecology

## Abstract

Leaf water content (LWC) has important physiological and ecological significance for plant growth. However, it is still unclear how LWC varies over large spatial scale and with plant adaptation strategies. Here, we measured the LWC of 1365 grassland plants, along three comparative precipitation transects from meadow to desert on the Mongolia Plateau (MP), Loess Plateau, and Tibetan Plateau, respectively, to explore its spatial variation and the underlying mechanisms that determine this variation. The LWC data were normally distributed with an average value of 0.66 g g^−1^. LWC was not significantly different among the three plateaus, but it differed significantly among different plant life forms. Spatially, LWC in the three plateaus all decreased and then increased from meadow to desert grassland along a precipitation gradient. Unexpectedly, climate and genetic evolution only explained a small proportion of the spatial variation of LWC in all plateaus, and LWC was only weakly correlated with precipitation in the water-limited MP. Overall, the lasso variation in LWC with precipitation in all plateaus represented an underlying trade-off between structural investment and water income in plants, for better survival in various environments. In brief, plants should invest less to thrive in a humid environment (meadow), increase more investment to keep a relatively stable LWC in a drying environment, and have high investment to hold higher LWC in a dry environment (desert). Combined, these results indicate that LWC should be an important variable in future studies of large-scale trait variations.

## Introduction

When plants emerged on land, access to water became a fundamental requirement for survival. Water is a key structural component of plants, allowing them to access light and exchange gases, especially in leaves. Water is also the main component of the cytoplasm and is essential for transporting substances. Furthermore, the water in leaves contributes to the processes of photosynthesis and respiration. Therefore, leaf water content (LWC, (fresh weight − drought weight)/fresh weight) should represent an important trait for plants that allows them to adapt to an altered environment at multiple levels, including the individual species, community, regional, and global levels. Water in leaves contributes to many processes in plants, yet LWC remains a focus of plant physiological studies only and is overlooked by large-scale studies^1–3^.

In nature, precipitation levels tend to represent a limiting resource for plants^4^, especially in arid and semi-arid regions^5^. Plant growth and development depend on the physiological processes of leaves to a large extent, including photosynthesis and transpiration^6,7^. In theory, to optimize function, leaves must maintain a relatively stable LWC^8^. LWC should remain relatively stable, show only small variations to adjust to changing environments or diurnal dynamics. This is because such stability is important during the process of evapotranspiration, where water loss is unavoidable at the leaf surface to uptake CO_2_^9^. LWC tends to be used as an auxiliary parameter in scientific research. Consequently, the significance of LWC, and associated adaptations, in response to the external environment remains unclear.

Plants in nature are subjected to extreme environmental conditions, including high temperature, drought, and low nutrient availability. Therefore, plants must develop protection mechanisms for LWC to balance costs and benefits. Hence, it is assumed that LWC is maintained at a relatively stable range, especially for leaves^10^. Through a combination of evolution and adaptation, LWC and its range in certain environments should be constant and predictable, similar to many other functional traits^11,12^. For instance, with increasing aridity, plants might regulate the acquisition of water to meet the requirements of the most limited resources. Plants always store large quantities of water resources, adjusting for water loss when sufficient water is available. In extreme drought environments, such as deserts, succulent plants tend to be prevalent, because of their higher water content and high storage capacity^13^. Thus, such plants likely cope better with periodic shortages in water, with stronger drought resistance^5,14–17^. Few studies on LWC have also been limited to fixed-point experiment or occur on small spatial scales. Plants in nature display long-term adaptations and responses to the environment. Plant growth and development depend on the basic physiological processes of leaves, in which water is not only a participant and product, but also an important solvent^6,7,18–20^. Thus, it is important to understand LWC and its variation over an environmental gradient when discussing the response of LWC to changing environment. Yet, knowledge on how LWC varies spatially along large-scale environmental gradients remains limited, along with the underlying mechanisms.

We hypothesized that, along a wet-dry gradient (i.e., from meadow to typical grassland to desert), LWC would first to drop and then rise owing to a combination of investment (survival strategies) and competition (between species) conditions. Fitness effects might exist in extreme, potentially lethal, environments^5^. To assess this hypothesis we explored the spatial variation and underlying mechanisms of LWC in plants at the biome scale. We consistently measured the LWC of 1300 grassland species on the Mongolia Plateau (MP), Loess Plateau (LP) and Tibetan Plateau (TP), along three comparative precipitation gradient transects, covering meadows, typical grasslands, and desert grasslands. However, these similar “natural control experiments” were subject to different primary limiting factors; namely, water limitation in MP, nutrient limitation in LP, and temperature limitation in TP. These differences provided a novel opportunity to explore spatial variation in LWC, and to verify our hypothesis on the effects of different limiting factors at the biome scale. The main objectives of this study were to: (1) explore spatial variation of LWC in grassland species on the main plateaus of the northern hemisphere; (2) demonstrate which factors influence LWC at the biome scale; and (3) discover the mechanisms underlying spatial variation along comparative transects. A combination of field-controlled experiments and the evaluation of multiple transects was used. Our results are expected to emphasize the importance of LWC as an important plant trait that should be incorporated into research studies.

## Results

### Spatial variation of LWC in grasslands

**Figure 1:**
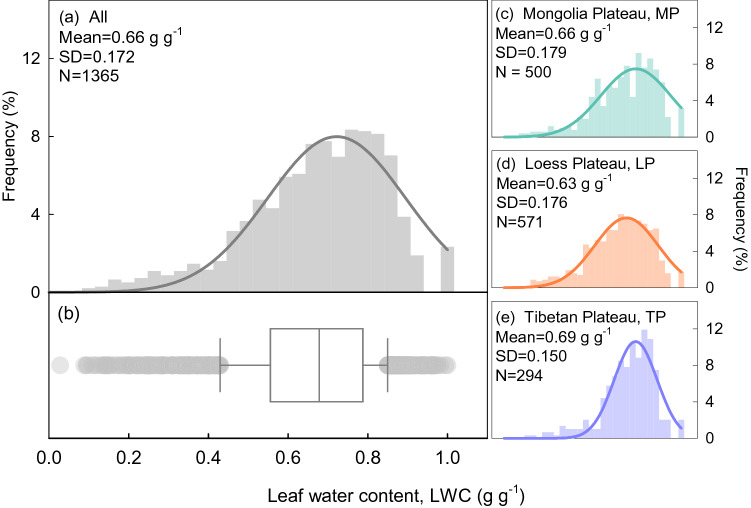
Frequency distribution of leaf water content (LWC) for 1365 species on three plateaus. Panels (**a**) and (**b**) present all species, and panels (**c**–**e**) present species from the Mongolia Plateau, Loess Plateau, and Tibetan Plateau, respectively.

The LWC of grassland plants on the three plateaus were normally distributed, with a mean of 0.66 ± 0.17 g g^−1^ (Fig. 1). The LWC of the three plateaus was highest on the TP (0.69 ± 0.15 g g^−1^), followed by MP (0.66 ± 0.18 g g^−1^) and LP (0.63 ± 0.18 g g^−1^); however, the difference was not significant (Fig. 1). The LWC of *Gramineae* was significantly lower than that of *Legumes* and other groups (Table 1).

**Table 1:** Changes to leaf water content (LWC, g g^−1^) among different plant life forms and different grassland types.

Meadow	Typical grassland	Desert grassland	Legume	Gramineae	Others
**Mongolia plateau (MP)**					
LWC					
0.699 ± 0.135^Aa^	0.594 ± 0.197^Ab^	0.766 ± 0.152^Ac^	0.601 ± 0.157^Aa^	0.541 ± 0.167^Ab^	0.701 ± 0.169^Ac^
**Loess plateau (LP)**					
LWC					
0.620 ± 0.188^Ba^	0.634 ± 0.165^Ba^	0.643 ± 0.182^Ba^	0.598 ± 0.155^Aa^	0.457 ± 0.171^Bb^	0.672 ± 0.156^Bc^
**Tibetan plateau (TP)**					
LWC					
0.674 ± 0.162^Aa^	0.687 ± 0.136^Cab^	0.728 ± 0.130^Ab^	0.711 ± 0.101^Ba^	0.560 ± 0.139^Ab^	0.702 ± 0.148^Aa^

^†^Significant differences were tested at *P* = 0.05, and different lowercase letters indicate significant differences. Lowercase letters compare different grassland types on the same plateau, while capital letters compare grassland types on different plateaus.

**Figure 2:**
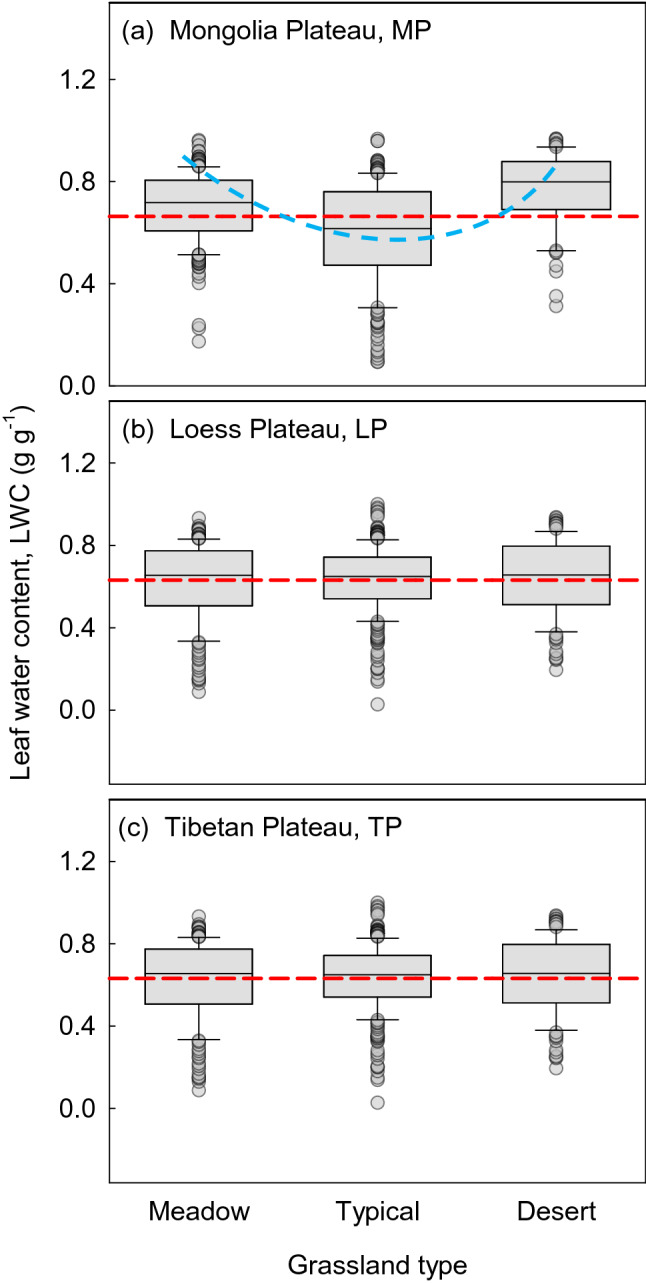
Changes to leaf water content (LWC) in different grassland types. Panels (**a**), (**b**) and (**c**) show the LWC on the Mongolia Plateau (MP), Loess Plateau (LP), and Tibetan Plateau (TP). Red dotted lines present the mean in each plateau, and blue dotted lines present virtual trends. Figures were done by SigmaPlot version 10.0.

Significant differences in LWC were observed among the grassland types (*P* < 0.05, Table 1), being ordered as typical grassland (0.594 g g^−1^) < meadow (0.699 g g^−1^) < desert grassland (0.766 g g^−1^) on the MP. The LWC was ordered as meadow (0.647 g g^−1^) < typical grassland (0.687 g g^−1^) < desert grassland (0.728 g g^−1^) (Fig. 2) on the TP, in with the LWC between meadow and desert grassland differing significantly (*P* < 0.05). However, no significant differences were found among the three grassland types for the LP. Gramineae had significantly lower LWC compared to other plant life forms, irrespective of transects (Table S2, *P* < 0.05).

### Regional variation of LWC and the factors that influence it

**Figure 3:**
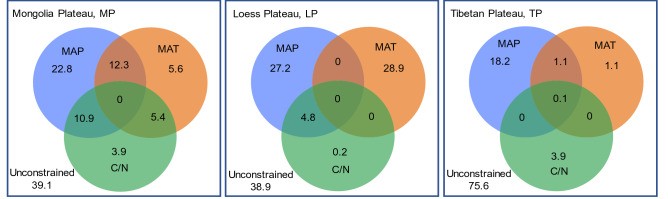
Results of redundancy analysis between leaf water content (LWC) and influencing factors. Numbers in circles represent the interpretation rate of the factor for LWC. MAT, mean annual temperature; MAP, mean annual precipitation; C/N, C/N ratio of soil.

**Figure 4:**
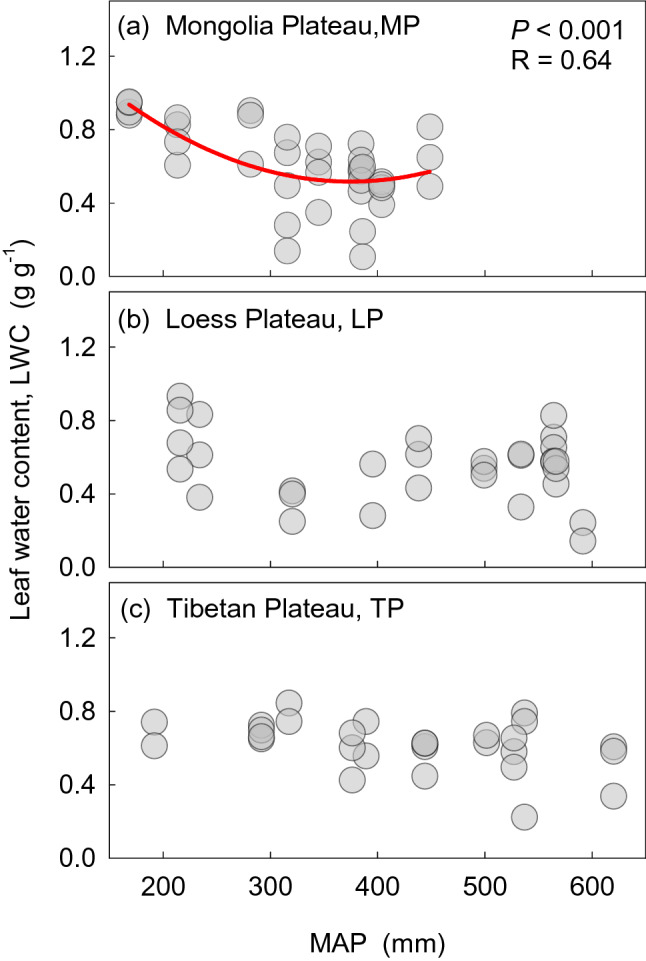
Relationship between leaf water content (LWC) and mean annual precipitation (MAP). Panels (**a**), (**b**) and (**c**) show the LWC correlated with MAP on the Mongolia Plateau (MP), Loess Plateau (LP), and Tibetan Plateau (TP). Figures were done by SigmaPlot version 10.0.

For dominant species, the correlation between LWC and environmental factors was relatively lower (Fig. S1). The RDA (Fig. 3) showed that the average interpretation rate of mean annual precipitation (MAP) for LWC was about 20% in each transect. Simple linear regression did not capture the relationships between LWC and precipitation well (Fig. 4). The LWC on the MP first declined, and then increased with precipitation from wet to dry, and was significant (R = 0.64, *P* < 0.001). Weak relationships were detected between LWC and precipitation on the LP and TP. When considering all species, the Pearson correlation coefficient matrix and the analyses of RDA both showed a weak relationship between LWC and environmental factors (Fig. S2 and S3).

The phylogenetic tree of LWC showed that the K value of all groups was less than 1. The significance test showed that the *P* value was greater than 0.05. Thus, phylogenetic time only had a small influence on the LWC of grassland plants on all plateaus (Table S3).

### Variation of LWC in grassland communities

**Figure 5:**
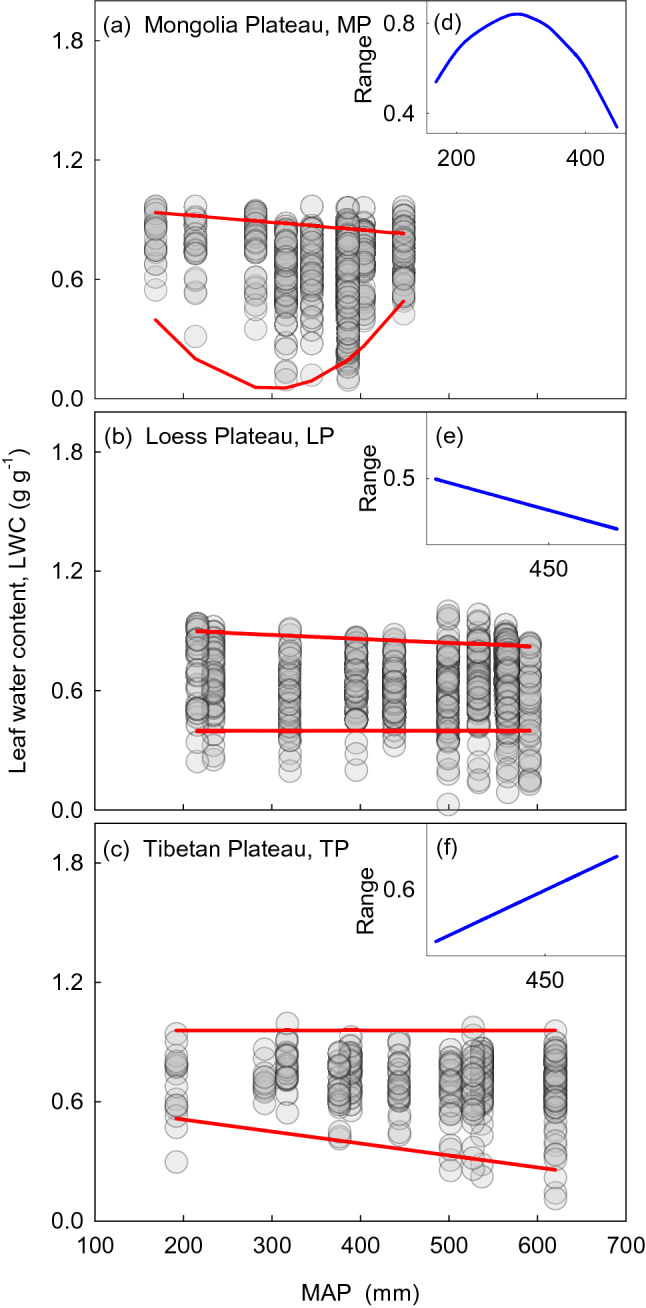
Range of leaf water content (LWC) in the community correlated with mean annual precipitation (MAP). Panels (**a**), (**b**) and (**c**) show the LWC in the community correlated with MAP in the Mongolia Plateau (MP), Loess Plateau (LP), and Tibetan Plateau (TP), respectively. Solid red lines indicate the linear regression analyses of 95% and 5% boundaries. Panels (**d**), (**e**), and (**f**) show the range of variation range in LWC with rainfall on the MP, LP and TP, respectively. Figures were done by SigmaPlot version 10.0.

Based on 95% quantile and 5% quantile regression analyses, the moisture gradient was significantly associated with the discrete degree of LWC. The boundaries showed a significant linear correlation with precipitation (Table 2). On the MP, it was significant at the two quantiles (Fig. 5a,d). On the LP, it was significant in the 95% quantile regression analyses (Fig. 5b,e). On the TP, it was significant in the 5% quantile regression analyses (Fig. 5c,f). The range of LWC in the three transects exhibited different relationships which were represented by different fitted linear regressions. (Fig. 5, Table 2).

**Table 2:** Boundary analyses and significance tests for the spatial variation of leaf water content.

Leaf water content (LWC)	Quantiles	Fitted equations	Significance
Mongolia plateau (MP)	95th	LWC = − 0.00038MAP + 1.00	*P* < 0.001
	5th	LWC = 0.00002MAP^2^ − 0.012MAP + 1.85	*P* < 0.001
	Range	LWC = − 0.00002MAP^2^ − 0.01162MAP − 0.85	–
Loess plateau (LP)	95th	LWC = − 0.0002MAP + 0.94	*P* < 0.001
	5th	LWC = 0.3980	–
	Range	LWC = − 0.0002MAP + 0.5420	–
Tibetan plateau (TP)	95th	LWC = 0.9575	–
	5th	LWC = − 0.00061MAP + 0.63	*P* < 0.001
	Range	LWC = 0.00061 + 0.3275	–

^†^95th and 5th represented 95% upper boundary and 5% lower boundary. Range is the fluctuation to leaf water content (LWC) between the upper and lower boundaries.

## Discussion

### LWC is a relatively stable trait representing the adaptations and responses of plants

**Figure 6:**
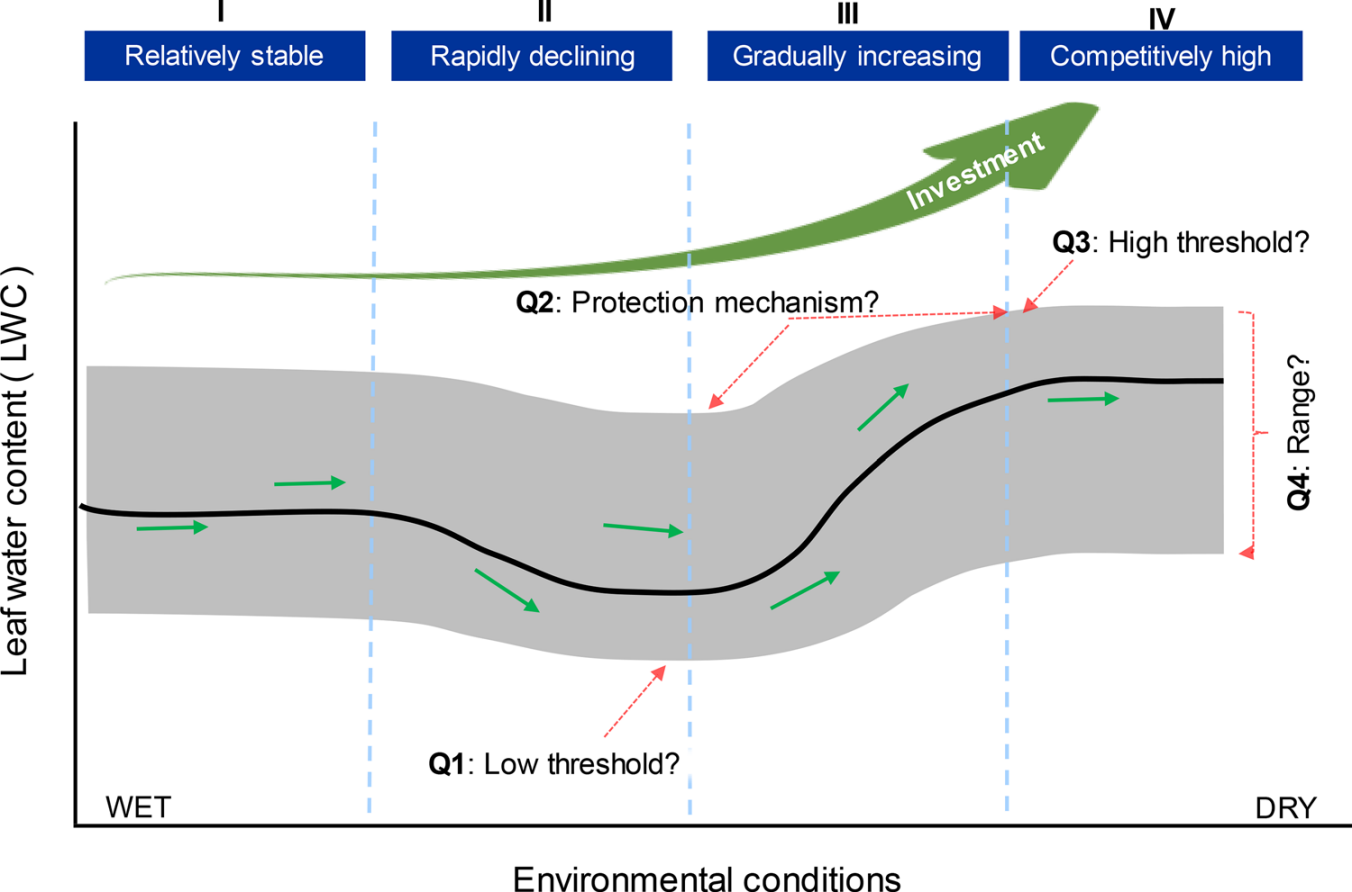
Underlying mechanism for spatial variation in leaf water content (LWC) among environmental gradients (investment vs. competition mechanism). LWC was relatively stable, with plants that are stabilized with low investment for water retention (I); LWC declined rapidly and stabilized, with the water conservation mechanism gradually forming to enhance moisture retention, while investment gradually increased (II); LWC gradually increased as a result of high investment and community competitive advantage (III); in phase IV, plants built the highest LWC with the highest investment. Four questions (Q1–Q4) require exploring in the future: What are the two thresholds in natural communities? What is the real investment of plants and how is it realized (e.g., stomata, anatomy, solutes)? What is the significance for the spectrum of the distribution frequency of LWC?

The LWC was relatively stable potentially representing the adaptations and responses of plants^13^. The LWC of live leaves was relatively consistent and comparable, providing a basis from which to investigate this parameter at large scales, as with other traits^12,21^. We suggested that the live leaves of plants maintain a relatively stable LWC under extreme drought, but at the cost of most leaves dying or falling to facilitate survival until an opportunity for recovery arises^5^. Thus, our results provided evidence that the distribution of LWC appeared nearly identical in all three plateaus (Fig. 6). Although plants make minor sacrifices to safeguard survival under limited or stressed environments: similar trade-offs are common for other plant traits and are termed ecological strategies^21,22^. Considering the importance of LWC on plant physiological, physical, and ecological functions, the relatively stable LWC of live leaves should be considered as an important trait.

### Plants in water deficient areas have higher LWC

Plants in water deficient regions have higher LWC, irrespective of the plateau on which they grow. Our study showed that the LWC of desert plants was higher than that of other grassland types especially the meadow grassland (Fig. 2), and this difference was significant for MP and TP. Water availability is essential for the survival and development of plants. To optimize the functioning of plants, for maintenance or survival, relatively stable LWC results from long-term evolution and adaptation, from the perspective of physiology or ecology.

Usually, plants in arid ecosystems form fleshy leaves, with thicker leaf cuticles, and shorter stomatal opening times^10,21,22^. Particular forms and structures of the leaves are better adapted to adverse conditions. The LWC of plants decreased and then increased from meadow to typical grassland and desert grasslands along the precipitation gradient especially in a precipitation limited environment such as the MP (Fig. 2). The lasso variation of LWC, from wet to dry, might be related to the investment of certain traits by plants. Two control systems may exist for the two grassland types with relatively higher water content from our results. In high moisture environments, plants are free to grow and develop without water limitation. Thus, plants do not need to invest much to maintain LWC^23,24^. In contrast, plants must maintain appropriate LWC in extreme drought environments to safeguard physiological activities. This phenomenon is achieved by increasing investment to maintain a relatively stable LWC by auxiliary organization (e.g., thick cuticle). Consequently, plants in arid areas have evolved diverse structures to avoid water loss, with the result that plants occupying desert grasslands have higher LWC. However, when water is scarce, plants must consume more energy to access or retain LWC, probably by regulating the structure and function of plants, or through community structure. Our results showed that LWC is barely connected with the climate gradient, except in arid areas (Fig. 4). Under conditions of limited precipitation, plants should invest more in physiological functioning, and reduce investment as precipitation increases along a gradient.

### Precipitation has a small effect on LWC in natural communities at large scales

Although climatic factors are guaranteed to affect variation in traits^25,26^, the effect of environment on LWC tends to be overestimated, to some extent. The Pearson correlation and RDA analyses showed that environmental factors only exerted a weak influence on the LWC of grassland plants on the three plateaus (Figs. 3, S1, S2, and S3). In all three transects, along a decreasing precipitation gradient from meadow to desert, a simple linear regression failed to capture the relationships between LWC and precipitation, except for a weak link on the MP (Fig. 4). Our results demonstrated that precipitation only had a small effect on LWC in natural communities at a large scale, which contrasted with our predictions. Our study found that lasso variation might occur in areas with water limitation (Fig. 4). Previous studies showed that species that experienced greater damage from heatwaves and stress are more sensitive to changing environment than species that experienced less heatwave damage and stress^27^. Plant LWC was more responsive to water changes in the water limitation region (MP).

Evolutionary history is considered to be a profound factor influencing plant functional traits at the biome scale^28–33^. However, LWC was minimally affected by phylogeny in this study (Table S3); instead, LWC appeared to be regulated by extreme environmental conditions, such as extreme drought. Community structure adjusts with the changes to the environment, with the “range” in LWC changing with the shift in community structure across environments. Plant species are hypothesized to differ with respect to range size, partly because of differences in environmental tolerance^34,35^. In communities that are restricted by different factors, the “range” changes in different ways. For instance, water was the main restrictive environment factor on the MP, and strongly influenced changes to LWC, as well as its distribution. The LWC evolved and adjusted strictly in accordance with the physiological requirements of water. In comparison, soil nutrients were the limiting factor on the LP (Fig. 5b,e). Changes to LWC were relatively weak and were affected by the higher boundary at which plants had higher LWC. Temperature was the main limiting factor on the TP (Fig. 5c,f), with the lower boundary of plants that had low LWC being affected by changes to the “range.” Therefore, plants with high LWC have better resistance to lower temperatures. When extreme temperatures occur, the community might adopt a sacrifice stratagem to eliminate species with high input and low income to ensure the energy balance of the community. Thus, shifting climates and more extreme climate events are expected to have a stronger negative impact on the performance and habitat suitability of species with small, rather than large, range sizes^27,36–38^.

### Underlying mechanisms in the spatial variation of LWC at the biome scale

Although LWC was weakly correlated with precipitation gradients at the biome scale, plant communities had similar investment-return strategies of LWC across plateaus that had different main limiting factors. These strategies arise as a consequence of trade-offs between resource acquisition and survival, as documented for the leaf economic spectrums and other traits^39–41^. Interestingly, lasso variation of LWC with a decreasing precipitation gradient was recorded on all plateaus. This phenomenon is explained by the fact that, in moist environments, plants do not need to invest in the water-retaining properties of leaves. As a result, LWC initially decreased with precipitation gradient in meadows. However, conservative investment has a base line, represented by the minimum LWC for normal photosynthesis, respiration and other functions. Consequently, with increasing drought pressure along the gradient, the LWC would drop below this tolerance threshold, with plants needing to increase investment to survive. As a result, LWC was lower, but relatively stable, in typical grasslands. In such areas, plants might develop certain traits, such as the thickening of cuticle and fleshy leaves, which are beneficial for water retention^10^. With increasing drought conditions (i.e., in deserts), most plants cannot survive, and eventually die, unless they have special mechanisms to retain moisture, which require the highest investment.

When plants live in drought for long periods of time, they adapt to the environment they are in. Thus, precipitation can reflects the relatively stable environment state of a region, and it is possible to compare different regions on a large scale. Compared with the Loess Plateau and the Tibet Plateau, the Mongolia Plateau is arid. Therefore, precipitation is the most intuitive representation of this arid environment, which is the main reason why we chose MAP. The relationship between LWC and decreasing precipitation gradients could be divided into four stages, as shown in Fig. 6. In stage I, LWC is relatively stable, with plants showing a low investment for water retention. Even if the LWC of plants decreases with decreasing precipitation, to some extent, it does not affect individual or community structure. In stage II, LWC declines rapidly and stabilizes, with a water conservation mechanism gradually forming to enhance the ability of plants to retain moisture, with a gradual increase in investment. When LWC decreases to the minimum tolerance threshold, some species with high investment and low income are lost, whereas species with low investment and high income are beneficial for maintaining the community. In stage III, LWC gradually increases as a result of high investment and the competitive advantage of the community. Some plants invest highly in water conservation and become dominant through competition. In comparison, some plants are lost, or lose dominance, because of a lower ability to retain moisture. In stage IV, plants have the highest LWC with the highest investment. When it is extremely dry, only plants with higher LWC, or a special ability to retain moisture, can survive. The community structure remains relatively stable, but simple.

Figure 6 demonstrates the importance of exploring the adaptive mechanisms of plants to changing environments in the future. What are the two thresholds (high threshold and low threshold) in natural communities? What is the actual investment of plants and how do they realize it (e.g., through regulating stomata, anatomy, solutes)? What is the significance for the spectrum regarding the distribution frequency of LWC in natural communities? Through analyzing LWC in combination with environmental information, research on plant traits can be expanded. Understanding how LWC responds to the environmental gradient and how mechanisms vary could help to develop new research directions and concepts on more complex communities. Therefore, we believe that LWC is important and could enhance analyses on the responses and adaptations of community structure to changing environments.

## Conclusions

Leaf water content (LWC) plays an important role in plant physiological and ecological functions. The three grassland transects surveyed in the northern hemisphere showed that the LWC of live leaves is relatively stable, with LWC in water restricted regions being higher and significantly responding to the precipitation gradient. Unexpectedly, precipitation and genetic evolution had weak effects on LWC. Lasso variation of LWC with a decreasing precipitation gradient was documented on all three plateaus. Plants exhibited similar investment-return strategies for LWC across plateaus with different main limiting factors. This phenomenon was the consequence of trade-offs between resource acquisition and survival. Our results provide new insights on the mechanisms underlying variability in LWC in response to environmental gradients among plant species. It is necessary to obtain more data on the specific range, threshold, and investment inflection points of the regulating mechanisms in future. Considering the importance of LWC on plant physiological, physical, and ecological functions, the relatively stable LWC of live leaves should be considered as an importance trait in future studies conducted at large scales, especially when exploring the responses and adaptations of plants to climate change.

## Materials and methods

### Study site

**Figure 7:**
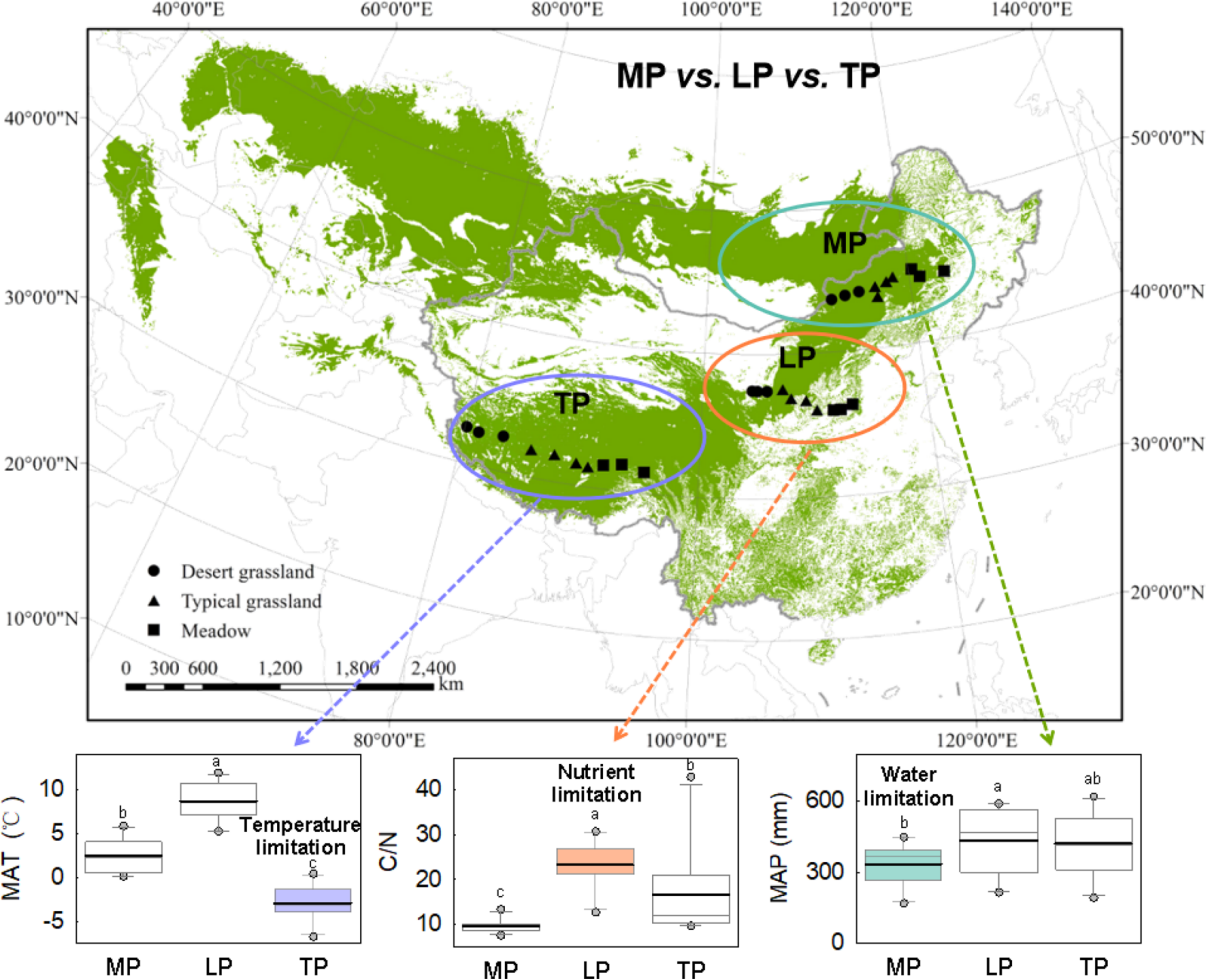
Experimental design and spatial distribution of sampling sites. We established three comparative transects in the Mongolia Plateau (MP), Loess Plateau (LP), and Tibetan Plateau (TP). These regions have different primary limitations for plant growth, representing meadow, typical grassland, and desert grassland, respectively. These comparative transects are expected to provide information on spatial variation in leaf water content (LWC) and the underlying mechanisms that influence it. The land cover data used in our study were collected from the Land Cover Type Climate Modelling Grid (CMG) product (MCD12C1) in 2012 (LP DAAC; https://lpdaac.usgs.gov). Figures were done by ERSI ArcGIS software (Version 10.1; Redlands, CA, USA).

Based the spatial distribution of grasslands in the northern hemisphere, we selected three major plateaus to conduct our experiment: namely the Mongolia Plateau (MP), Loess Plateau (LP), and Tibetan Plateau (TP) (Fig. 7). The MP, LP, and TP cover the largest extent of Eurasian grasslands, and encompass the world’s largest loess deposition area, and the highest alpine grasslands. Consequently, these areas provide the opportunity to conduct unique but similar “natural controlled experiments” to explore spatial variation in LWC and influencing factors at the biome scale.

We setup precipitation gradient transects from east to west, covering meadows, typical grassland, and desert grasslands on the MP, LP, and TP (Fig. 7). This approach was used because transects are economical and scientific, as stipulated by the International Geosphere-Biosphere Programme (IGBP)^42^. Mean annual precipitation (MAP), mean annual temperature (MAT), and soil carbon and nitrogen ratio (C:N ratio) of the three plateaus were significantly different (Fig. 7). MAP was lowest on the MP, C:N ratio was highest in LP, and MAT was lowest on the TP. Based on previous studies^43–45^, we assumed that different primary limiting factors operate at MP, LP and TP, which were, respectively, environmental water limitation (water shortage), soil nutrient limitation (low nutrient), and environmental limitation (low temperature) (Fig. 7). Thus, the three transects were regulated by different factors, despite being set up along a similar precipitation gradients. Gramineae and legumes were the dominant species in most of the sampling sites. The specific families and quantities of plant samples are shown in Table S1.

Each transect spanned more than 1000 km. Ten sites were established from east to west based on the spatial distribution types of grasslands for each plateaus. MAT ranged from 0.1 to 5.8 °C on the MP, from 5.2 to 11.8 °C on the LP, and—6.8 to 0.4 °C on the TP (Table S4). MAP ranged from 168.3 to 448.5 mm on the MP, 215.5 to 591.2 mm in LP, and from 191.7 to 619.9 mm in TP (Table S4). Climate data including MAP and MAT were derived from the interpolation of records from meteorological stations across China during 1961–2010.

### Field sampling

First, we set up eight 1 m × 1 m quadrats at each site along the three transects. We investigated the species composition of the community and above-ground biomass for each plant species^12^. Then, we collected leaf samples from all plant species in a 1 km × 1 km area to measure LWC. The plants in our study were identified in the field by senior plant taxonomists, and the plant species were further revised and proofread by Flora Reipublicae Popularis Sinicae (http://www.iplant.cn/frps). Previous studies demonstrated that using dominant species alone is not sufficient to make objective interpretations^46^. Our approach may have allowed us to analyze the data from different perspectives across all species.

Field sampling was conducted from mid-July to late August in 2018. Collected plant leaves were refrigerated with an ice-bag and transferred to the laboratory within two hours. The LWC of complex leaves for each species was then measured in the laboratory (three replicates). Following Cornelissen et al. (2003)^47^ the fresh weight of leaves (FW) was first measured using an analytical balance. Then, the leaves were transferred to the oven to a constant weight for 48 h at 70 °C. Then, drought weight (DW) was measured, and LWC was calculated as (Eq. 1):

1 $$LWC=\frac{FW-DW}{FW} \left({\mathrm{g} \mathrm{g}}^{-1}\right)$$

Dominant species were identified using the relative-importance value, which is a combination of the relative frequency, relative number, and relative coverage of each plant species in the eight 1 m × 1 m quadrats. The plant species of each sample point was considered as dominant species when the importance value exceeded 10%^48,49^. Overall, we measured the LWC of 1365 plant species along the three transects.

### Construction of the phylogenetic tree and calculation of the phylogenetic signal K value

To explore how evolution has influenced LWC, the Plant List was used to confirm species names (htQTP://www.theplantlist.org). Then “S.PhyloMaker” was used to generate a phylogeny^50^. We calculated Blomberg’s K^51^ and its significance using the ‘Picante’ package in R. Then, we evaluated the strength of the phylogenetic signal of LWC for each species in families containing more than 10 species.

### Data analyses

The LWC of plant species from specific sites was represented as mean ± SD. Variation to LWC across sites and transects were compared using one-way analyses of variance (ANOVA). The relationships between LWC and environmental factors was assessed using Pearson’s correlation coefficient. The interpretation rate of the environmental factors (MAT, MAP, C:N ratio) to LWC was explored using redundancy analyses (RDA), with pure and joint effects being calculated. Our data passed the normality test. Quadratic regression analysis (with lower AIC compared to linear regression) was used to explore the relationship between MAP and LWC using SigmaPlot version 10.0, from Systat Software, Inc., Point Richmond, CA, www.systatsoftware.com. Data analyses were conducted using SPSS 13.0 (Chicago, IBM Corp, USA) and R (version 3.14.3, R Development Core Team, Vienna, Austria, 2012, https://www.R-project.org). Graphs were drawn using SigmaPlot (Systat Software, Point Richmond, CA), and ERSI ArcGIS software (Version 10.1; Redlands, CA, USA).

### Additional information

We state that all plant materials involved in the study were collect with the permission of the Institute of Geographic Sciences and Natural Resources Research and the local authorities. Our study protocol comply with relevant institutional, national, and international guideline and legislation. We would like to express our heartfelt thanks to the following four researchers for their contributions to our research: Professor Huanhu Dong (Shanxi Agricultural University), professor Wenjun Gao (Shanxi Agricultural University), professor Yaozhi Zhou (Tebit University) and professor Chuantao Song (Northeast Normal University).” Professor Dong and professor Gao have been engaged in plant classification in the Loess Plateau region for many years, and have a full understanding of the plants in the Loess Plateau. Professor Song has long been engaged in plant research in the grasslands of northern China, especially in the Mongolian Plateau region, and has rich experience in plant identification. Professors Zhou has been engaged in the research of alpine grassland for many year, and has carried out grassland surveys on Tibetan Plateau for many times, so he has very rich experience in identifying plants. We did not deposit the voucher specimen of these materials in a publicly available herbarium.

## Supplementary Information

Supplementary Information

## Data Availability

All data for this paper are included in the manuscript and supporting information.

## References

[1] Díaz S, Hodgson JG, Thompson K, Cabido M, Zak MR (2010). The plant traits that drive ecosystems: evidence from three continents. J. Veg. Sci..

[2] He N (2019). Ecosystem traits linking functional traits to macroecology. Trends Ecol Evol.

[3] Reich PB, Lusk WCH (2007). Predicting leaf physiology from simple plant and climate attributes: a global GLOPNET analysis. Ecol. Appl..

[4] Shi P, Preisler HK, Quinn BK, Zhao J, Hlscher D (2020). Precipitation is the most crucial factor determining the distribution of moso bamboo in Mainland China. Global Ecol. Conserv..

[5] Bassirirad GH (2003). Extreme events as shaping physiology, ecology, and evolution of plants: toward a unified definition and evaluation of their consequences. New Phytol..

[6] Boyer JS (1985). Water transport. Annu. Rev. Plant Physiol..

[7] Kromer S (1995). Respiration during photosynthesis. Annu. Rev. Plant Physiol. Plant Mol. Biol..

[8] Carl VJ, VanLoocke A (2015). Terrestrial ecosystems in a changing environment: a dominant role for water. Annu. Rev. Plant Biol..

[9] Heinen RB, Qing Y, François C (2009). Role of aquaporins in leaf physiology. J. Exp. Bot..

[10] Chapin FS, Matson PA, Mooney HA (2011). Principles of Terrestrial Ecosystem Ecology.

[11] Ma Z (2018). Evolutionary history resolves global organization of root functional traits. Nature.

[12] Zhang J (2017). C:N: P stoichiometry in China's forests: from organs to ecosystems. Funct. Ecol..

[13] Grime JP (1977). Evidence for the existence of three primary strategies in plants and its relevance to ecological and evolutionary theory. Am. Nat..

[14] Bassirirad H, Caldwell MM (1992). Root growth, osmotic adjustment and NO3-uptake during and after a period of drought in Artemisia tridentata. Aust. J. Plant Physiol..

[15] Bassirirad H, Caldwell MM (1992). Temporal changes in root growth and 15N uptake and water relations of two tussock grass species recovering from water stress. Physiol. Plant..

[16] Bassirirad H (1999). Short-term patterns in water and nitrogen acquisition by two desert shrubs following a simulated summer rain. Plant Ecol..

[17] Gebauer RLE, Ehleringer JR (2000). Water and nitrogen uptake patterns following moisture pulses in a cold desert community. Ecology.

[18] Liu M, Niklas KJ, Niinemets L, Hlscher D, Shi P (2020). Comparison of the scaling relationships of leaf biomass versus surface area between spring and summer for two deciduous tree species. Forests.

[19] Shi P, Li Y, Hui C, Ratkowsky DA, Niinemets L (2019). Does the law of diminishing returns in leaf scaling apply to vines? Evidence from 12 species of climbing plants. Glob. Ecol. Conserv..

[20] Yu X, Hui C, Sandhu HS, Lin Z, Shi P (2019). Scaling relationships between leaf shape and area of 12 Rosaceae species. Symmetry.

[21] Liu C (2017). Variation of stomatal traits from cold temperate to tropical forests and association with water use efficiency. Funct. Ecol..

[22] Am H, Fi W (2003). The role of stomata in sensing and driving environmental change. Nature.

[23] Huang W, Ratkowsky DA, Hui C, Wang P, Shi P (2019). Leaf fresh weight versus dry weight: which is better for describing the scaling relationship between leaf biomass and leaf area for broad-leaved plants?. Forests.

[24] Huang W, Reddy GV, Li Y, Larsen JB, Shi P (2020). Increase in absolute leaf water content tends to keep pace with that of leaf dry mass—evidence from bamboo plants. Symmetry.

[25] Yang Y (2019). Quantifying leaf-trait covariation and its controls across climates and biomes. New Phytol..

[26] Huang W, Fonti P, Rbild A, Larsen JB, Hansen JK (2021). Variability Among Sites and Climate Models Contribute to Uncertain Spruce Growth Projections in Denmark. Forests.

[27] Aspinwall MJ (2019). Range size and growth temperature influence Eucalyptus species responses to an experimental heatwave. Glob. Change Biol..

[28] Shao J (2019). Plant evolutionary history mainly explains the variance in biomass responses to climate warming at a global scale. New Phytol..

[29] He J, Reddy GV, Liu M, Shi P (2020). A general formula for calculating surface area of the similarly shaped leaves: evidence from six Magnoliaceae species. Glob. Ecol. Conserv..

[30] Guo X, Reddy GV, He J, Li J, Shi P (2020). Mean-variance relationships of leaf bilateral asymmetry for 35 species of plants and their implications. Glob. Ecol. Conserv..

[31] Shi P-J, Li Y-R, Niinemets Ü, Olson E, Schrader J (2020). Influence of leaf shape on the scaling of leaf surface area and length in bamboo plants. Trees.

[32] Shi P (2019). Leaf area–length allometry and its implications in leaf shape evolution. Trees.

[33] Yu X, Shi P, Schrader J, Niklas KJ (2020). Nondestructive estimation of leaf area for 15 species of vines with different leaf shapes. Am. J. Bot..

[34] Brown JH (1984). On the relationship between abundance and distribution of species. Am. Nat..

[35] Slatyer RA, Hirst M, Sexton JP (2013). Niche breadth predicts geographical range size: a general ecological pattern. Ecol. Lett..

[36] Gonzalez-Orozco CE (2016). Phylogenetic approaches reveal biodiversity threats under climate change. Nat. Clim. Change.

[37] Pacifici M (2015). Assessing species vulnerability to climate change. Nat. Clim. Chang..

[38] Thuiller W, Lavorel S, Araújo MB (2005). Niche properties and geographical extent as predictors of species sensitivity to climate change. Glob. Ecol. Biogeogr..

[39] Wright IJ (2007). Relationships among ecologically important dimensions of plant trait variation in seven Neotropical forests. Ann. Bot..

[40] Reich PB (2014). The world-wide ‘fast–slow’plant economics spectrum: a traits manifesto. J. Ecol..

[41] Kong D (2014). Leading dimensions in absorptive root trait variation across 96 subtropical forest species. New Phytol..

[42] Koch, G. W., Scholes, R. J., Steffen, W. L., Vitousek, P. M. & Walker, B. H. The IGBP terrestrial transects: science plan. *Global Change Report* (1995).

[43] Liu Z, Shao MA, Wang Y (2011). Effect of environmental factors on regional soil organic carbon stocks across the Loess Plateau region China. Agric. Ecosyst. Environ..

[44] Bai Y (2008). Primary production and rain use efficiency across a precipitation gradient on the Mongolia plateau. Ecology.

[45] Chen H (2013). The impacts of climate change and human activities on biogeochemical cycles on the Q inghai-T ibetan P lateau. Glob. Change Biol..

[46] Dee LE (2019). When do ecosystem services depend on rare species?. Trends Ecol. Evol..

[47] Cornelissen J (2003). A handbook of protocols for standardised and easy measurement of plant functional traits worldwide. Aust. J. Bot..

[48] Lamont BB, Downes S, Fox JE (1977). Importance–value curves and diversity indices applied to a species-rich heathland in Western Australia. Nature.

[49] Zhang T, Guo R, Gao S, Guo J, Sun W (2015). Responses of plant community composition and biomass production to warming and nitrogen deposition in a temperate meadow ecosystem. PLoS ONE.

[50] Qian H, Jin Y (2016). An updated megaphylogeny of plants, a tool for generating plant phylogenies and an analysis of phylogenetic community structure. J. Plant Ecol..

[51] Blomberg SP, Garland T, Ives AR (2003). Testing for phylogenetic signal in comparative data: behavioral traits are more labile. Evolution.

