# Supplementary material for: Spatial variation and mechanisms of leaf water content in grassland plants at the biome scale: evidence from three comparative transects
Source: Sci Rep. 2021 Apr 29;11:9281. doi: 10.1038/s41598-021-88678-7 (PMC8084930; doi:10.1038/s41598-021-88678-7)
Supplement: Supplementary file 1 — Supplementary Information [file 41598_2021_88678_MOESM1_ESM.docx]

**Supporting information**

**Spatial variation** **and mechanisms of leaf water content in grassland plants at the biome scale: Evidence from three comparative transects**

Ruomeng Wang^1,2^, Nianpeng He^1,2,3*^, Shenggong Li ^1,2*^,Li Xu^1^, Mingxu Li^1^,

^1^ Key Laboratory of Ecosystem Network Observation and Modeling, Institute of Geographic Sciences and Natural Resources Research, Chinese Academy of Sciences, Beijing, 100101, China

^2^ College of Resources and Environment, University of Chinese Academy of Sciences, Beijing, 100049, China

^3^ Key Laboratory of Vegetation Ecology, Ministry of Education, Changchun, 130024, China

*Correspondent author: Nianpeng He, Ph.D, Shenggong Li, Ph.D

Key Laboratory of Ecosystem Network Observation and Modeling, Institute of Geographic Sciences and Natural Resources Research, Chinese Academy of Sciences, 11A, Datun Road, Chaoyang District, Beijing 100101, China

Tel.: +86-10-64889263; Fax: +86-10-64889399

E-mail address: [henp@igsnrr.ac.cn](mailto:henp@igsnrr.ac.cn) (N. He)**,** [lisg@igsnrr.ac.cn](mailto:lisg@igsnrr.ac.cn) (S. Li)

**Table S1 Information of sampling species**

| Loess Plateau (LP) | |  | Mongolia Plateau (MP) | |  | Tibetan Plateau (TP) | |
| --- | --- | --- | --- | --- | --- | --- | --- |
| Family | number |  | Family | number |  | Family | number |
| *Amaranthaceae* | 26 |  | *Amaranthaceae* | 38 |  | *Amaranthaceae* | 2 |
| *Amaranthaceae* | 2 |  | *Amaranthaceae* | 4 |  | *Amaryllidaceae* | 1 |
| *Amaryllidaceae* | 19 |  | *Amaryllidaceae* | 49 |  | *Apiaceae* | 5 |
| *Apiaceae* | 4 |  | *Apiaceae* | 10 |  | *Araliaceae* | 2 |
| *Apocynaceae* | 2 |  | *Apocynaceae* | 1 |  | *Berberidaceae* | 1 |
| *Apocynaceae* | 19 |  | *Apocynaceae* | 4 |  | *Bignoniaceae* | 4 |
| *Bignoniaceae* | 6 |  | *Betulaceae* | 1 |  | *Boraginaceae* | 8 |
| *Brassicaceae* | 4 |  | *Boraginaceae* | 8 |  | *Brassicaceae* | 14 |
| *Campanulaceae* | 3 |  | *Brassicaceae* | 7 |  | *Caprifoliaceae* | 2 |
| *Caprifoliaceae* | 6 |  | *Campanulaceae* | 2 |  | *Caprifoliaceae* | 3 |
| *Caryophyllaceae* | 2 |  | *Caprifoliaceae* | 2 |  | *Caryophyllaceae* | 7 |
| *Celastraceae* | 3 |  | *Caprifoliaceae* | 1 |  | *Compositae* | 42 |
| *Compositae* | 90 |  | *Caryophyllaceae* | 4 |  | *Crassulaceae* | 4 |
| *Convolvulaceae* | 12 |  | *Compositae* | 62 |  | *Cyperaceae* | 18 |
| *Cupressaceae* | 3 |  | *Convolvulaceae* | 7 |  | *Elaeagnaceae* | 1 |
| *Cyperaceae* | 7 |  | *Crassulaceae* | 1 |  | *Euphorbiaceae* | 14 |
| *Ebenaceae* | 1 |  | *Cyperaceae* | 9 |  | *Fabaceae* | 35 |
| *Elaeagnaceae* | 4 |  | *Elaeagnaceae* | 2 |  | *Gentianaceae* | 7 |
| *Ephedraceae* | 1 |  | *Elaeagnaceae* | 1 |  | *Geraniaceae* | 1 |
| *Euphorbiaceae* | 9 |  | *Ephedraceae* | 3 |  | *Iridaceae* | 2 |
| *Fabaceae* | 68 |  | *Equisetaceae* | 2 |  | *Lamiaceae* | 12 |
| *Gentianaceae* | 5 |  | *Euphorbiaceae* | 5 |  | *Onagraceae* | 2 |
| *Geraniaceae* | 5 |  | *Fabaceae* | 55 |  | *Papaveraceae* | 3 |
| *Iridaceae* | 2 |  | *Iridaceae* | 14 |  | *Plantaginaceae* | 1 |
| *Juglandaceae* | 2 |  | *Lamiaceae* | 13 |  | *Poaceae* | 34 |
| *Lamiaceae* | 9 |  | *Linaceae* | 1 |  | *Polygonaceae* | 10 |
| *Lamiaceae* | 3 |  | *Moraceae* | 2 |  | *Polygonaceae* | 1 |
| *Linaceae* | 4 |  | *Papaveraceae* | 1 |  | *Primulaceae* | 3 |
| *Magnoliaceae* | 1 |  | *Papaveraceae* | 1 |  | *Ranunculaceae* | 6 |
| *Malvaceae* | 5 |  | *Pinaceae Lindl.* | 1 |  | *Rosaceae* | 26 |
| *Moraceae* | 4 |  | *Plantaginaceae* | 3 |  | *Rubiaceae* | 1 |
| *Pinaceae Lindl.* | 1 |  | *Plumbaginaceae* | 2 |  | *Salicaceae* | 3 |
| *Plantaginaceae* | 2 |  | *Poaceae* | 82 |  | *Saxifragaceae* | 1 |
| *Platanaceae* | 1 |  | *Polygalaceae* | 3 |  | *Scrophulariaceae* | 12 |
| *Plumbaginaceae* | 1 |  | *Polygonaceae* | 9 |  | *Solanaceae* | 2 |
| *Poaceae* | 95 |  | *Portulacaceae* | 3 |  | *Tamaricaceae* | 3 |
| *Polygalaceae* | 4 |  | *Primulaceae* | 2 |  | *Urticaceae* | 1 |
| *Polygonaceae* | 4 |  | *Ranunculaceae* | 11 |  |  |  |
| *Ranunculaceae* | 8 |  | *Rosaceae* | 31 |  |  |  |
| *Rhamnaceae* | 10 |  | *Rubiaceae* | 3 |  |  |  |
| *Rosaceae* | 27 |  | *Rutaceae* | 8 |  |  |  |
| *Rubiaceae* | 6 |  | *Salicaceae* | 4 |  |  |  |
| *Rutaceae* | 7 |  | *Scrophulariaceae* | 5 |  |  |  |
| *Salicaceae* | 10 |  | *Solanaceae* | 2 |  |  |  |
| *Scrophulariaceae* | 2 |  | *Ulmaceae* | 10 |  |  |  |
| *Scrophulariaceae* | 6 |  | *Urticaceae* | 1 |  |  |  |
| *Selaginellaceae* | 1 |  | *Vitaceae* | 1 |  |  |  |
| *Simaroubaceae* | 5 |  | *Zygophyllaceae* | 9 |  |  |  |
| *Solanaceae* | 8 |  |  |  |  |  |  |
| *Tamaricaceae* | 6 |  |  |  |  |  |  |
| *Thymelaeaceae* | 5 |  |  |  |  |  |  |
| *Ulmaceae* | 14 |  |  |  |  |  |  |
| *Violaceae* | 5 |  |  |  |  |  |  |
| *Zygophyllaceae* | 12 |  |  |  |  |  |  |

**Table S2** Significance difference test of leaf water content (LWC，g g^-1^) for all species in three plateaus.

|  |  | Transects |  |
| --- | --- | --- | --- |
|  | Mongolia plateau (MP) | Loess plateau (LP) | Tibetan Plateau (TP) |
| LWC | 0.66±0.179^a^ | 0.63±0.176^a^ | 0.69±0.150^a^ |
|  | Plant life form | | |
|  | Legume | Gramineae | Others |
| LWC | 0.62±0.152^A^ | 0.51±0.169^B^ | 0.69±0.160^A^ |

^†^ Small letters were used to compared for different plateau, and capital letters for different plant life forms of three transects. Gramineae and legumes are the dominant species of most semi-arid grassland. The sample sizes of legumes, gramineae and other plants were 157, 200 and 1008, respectively.

**Table S3** Phylogenetic signal K value and different test results (P < 0.05)

|  | Mongolia plateau (MP) | Loess plateau (LP) | Tibetan Plateau (TP) |
| --- | --- | --- | --- |
| K | 0.01347257 | 0.01555026 | 0.05635451 |
| P | 0.322 | 0.102 | 0.082 |
|  | Mongolia plateau (MP) | | |
|  | Meadow | Typical grassland | Desert grassland |
| K | 0.03675451 | 0.0746006 | 0.08643485 |
| P | 0.14 | 0.002 | 0.016 |
|  | Loess plateau (LP) | | |
|  | Meadow | Typical grassland | Desert grassland |
| K | 0.02499267 | 0.01590475 | 0.06844421 |
| P | 0.523 | 0.132 | 0.022 |
|  | Tibetan Plateau (TP) | | |
|  | Meadow | Typical grassland | Desert grassland |
| K | 0.08030684 | 0.02578338 | 0.2040529 |
| P | 0.21 | 0.791 | 0.033 |

^†^ The differences in plant traits are the result of long-term adaptation of plants to environmental heterogeneity. In addition, they are also significantly affected by the evolutionary history of species. woody plant leaf traits are significantly affected by the evolutionary history. In general, the difference in traits between closely related species are small, and the functional traits of distantly related species are quite different. While studying large-scale climate effects, it is necessary to consider Phylogenetic relationship, that is, whether the functional traits of the species are tested for phylogenetic signals, usually expressed in K, and there is also a measure of K: K >1 indicates that the functional traits are more evolved than the Brownian motion model, K <1 indicates that the functional traits exhibit a weaker phylogenetic signal than the Brownian motion model.

**Table S4** The basic information for the sample plots of grasslands in Mongolia Plateau, Loess Plateau and Tibetan Plateau, respectively

|  |  | Mongolia Plateau (MP) | | | | | | | | | | | |
| --- | --- | --- | --- | --- | --- | --- | --- | --- | --- | --- | --- | --- | --- |
|  |  | Meadow | | |  | Typical grassland | | | |  | Desert grassland | | |
| Site name |  | MP01 | MP02 | MP03 |  | MP04 | MP05 | MP06 | MP07 |  | MP08 | MP09 | MP10 |
| Longitude (°E) |  | 123.51 | 121.04 | 120.33 |  | 118.36 | 116.52 | 116.67 | 117.68 |  | 114.89 | 113.50 | 112.15 |
| Latitude (°N) |  | 44.59 | 44.52 | 45.11 |  | 44.77 | 44.26 | 43.55 | 44.51 |  | 44.01 | 43.84 | 43.63 |
| Altitude (m) |  | 144.00 | 269.00 | 660.00 |  | 1019.00 | 1129.00 | 1272.00 | 1024.00 |  | 1101.00 | 1022.00 | 955.00 |
| MAT (℃) |  | 5.10 | 5.80 | 3.72 |  | 0.56 | 1.17 | 0.16 | 1.96 |  | 0.10 | 2.47 | 3.69 |
| MAP (mm) |  | 448.47 | 384.39 | 384.47 |  | 403.58 | 315.49 | 385.81 | 344.68 |  | 281.18 | 213.16 | 168.29 |
| DI |  | 24.76 | 20.28 | 23.36 |  | 31.86 | 23.54 | 31.64 | 24.01 |  | 23.19 | 14.24 | 10.24 |
| Carbon of soil (%) |  | 0.88 | 0.85 | 2.52 |  | 1.45 | 1.76 | 2.12 | 2.15 |  | 1.00 | 0.43 | 0.21 |
| Nitrogen of soil (%) |  | 0.07 | 0.09 | 0.25 |  | 0.14 | 0.19 | 0.22 | 0.22 |  | 0.11 | 0.05 | 0.03 |
| C/N radio of soil |  | 13.30 | 9.32 | 10.25 |  | 10.14 | 9.34 | 9.46 | 9.85 |  | 8.79 | 8.46 | 7.62 |
|  |  | Loess Plateau (LP) | | | | | | | | | | | |
|  |  | Meadow | | |  | Typical grassland | | | |  | Desert grassland | | |
| Site name |  | LP01 | LP02 | LP03 |  | LP04 | LP05 | LP06 | LP07 |  | LP08 | LP09 | LP10 |
| Longitude (°E) |  | 113.36 | 112.29 | 111.64 |  | 110.18 | 109.24 | 107.92 | 107.19 |  | 105.78 | 104.92 | 104.44 |
| Latitude (°N) |  | 36.29 | 35.99 | 35.99 |  | 36.07 | 36.74 | 36.93 | 37.58 |  | 37.42 | 37.44 | 37.46 |
| Altitude (m) |  | 804.00 | 894.00 | 833.00 |  | 966.00 | 1268.00 | 1383.00 | 1535.00 |  | 1293.00 | 1378.00 | 1714.00 |
| MAT (℃) |  | 11.85 | 9.96 | 10.66 |  | 10.72 | 9.50 | 7.46 | 5.23 |  | 5.87 | 7.56 | 7.71 |
| MAP (mm) |  | 563.88 | 591.18 | 566.12 |  | 533.36 | 498.89 | 438.09 | 395.14 |  | 320.35 | 233.88 | 215.50 |
| DI |  | 21.51 | 24.68 | 22.84 |  | 21.45 | 21.32 | 20.90 | 21.63 |  | 16.82 | 11.10 | 10.14 |
| Carbon of soil (%) |  | 2.42 | 1.82 | 3.17 |  | 2.63 | 2.37 | 2.31 | 1.37 |  | 1.68 | 1.09 | 1.54 |
| Nitrogen of soil (%) |  | 0.13 | 0.14 | 0.14 |  | 0.11 | 0.09 | 0.10 | 0.06 |  | 0.06 | 0.04 | 0.07 |
| C/N radio of soil |  | 18.82 | 12.72 | 22.86 |  | 24.32 | 26.63 | 22.71 | 23.57 |  | 26.64 | 30.64 | 21.93 |
|  |  | Tibetan Plateau (TP) | | | | | | | | | | | |
|  |  | Meadow | | |  | Typical grassland | | | |  | Desert grassland | | |
| Site name |  | TP01 | TP02 | TP03 |  | TP04 | TP05 | TP06 | TP07 |  | TP08 | TP09 | TP10 |
| Longitude (°E) |  | 95.45 | 93.53 | 92.01 |  | 90.74 | 89.72 | 87.82 | 85.84 |  | 83.34 | 81.23 | 80.15 |
| Latitude (°N) |  | 31.46 | 31.85 | 31.64 |  | 31.38 | 31.54 | 31.87 | 31.92 |  | 32.41 | 32.30 | 32.48 |
| Altitude (m) |  | 4104.00 | 4509.00 | 4587.00 |  | 4617.00 | 4588.00 | 4570.00 | 4938.00 |  | 4578.00 | 4558.00 | 4328.00 |
| MAT (℃) |  | 0.41 | -1.50 | -4.37 |  | -6.76 | -3.06 | -2.57 | -3.77 |  | -3.90 | -3.49 | -1.27 |
| MAP (mm) |  | 619.86 | 536.77 | 501.38 |  | 526.89 | 443.79 | 389.09 | 376.17 |  | 317.13 | 291.42 | 191.71 |
| DI |  | 49.63 | 52.63 | 74.23 |  | 135.50 | 53.29 | 43.63 | 50.30 |  | 43.34 | 37.32 | 18.30 |
| Carbon of soil (%) |  | 4.73 | 4.63 | 5.12 |  | 0.82 | 2.01 | 1.74 | 4.23 |  | 0.85 | 0.55 | 0.34 |
| Nitrogen of soil (%) |  | 0.45 | 0.41 | 0.40 |  | 0.08 | 0.12 | 0.07 | 0.10 |  | 0.08 | 0.03 | 0.03 |
| C/N radio of soil |  | 10.47 | 11.38 | 12.84 |  | 9.75 | 16.10 | 24.03 | 42.80 |  | 10.09 | 19.99 | 10.34 |

^†^ MAT, mean annual temperature; MAP, mean annual precipitation; DI, drying index.

**Fig.S1** Pearson correlation matrix of dominant species. Blue indicates positive correlation, red indicates negative correlation, “×” indicates that the difference test is not significant. MAT, mean annual temperature; MAP, mean annual precipitation; DI, drying index; C, carbon of soil (%); N, nitrogen of soil (%); CN, C/N radio of soil; LWC, leaf water content.

**Fig.S2** Pearson correlation matrix of all species. Blue indicates positive correlation, red indicates negative correlation, “×” indicates that the difference test is not significant. MAT, mean annual temperature; MAP, mean annual precipitation; DI, drying index; C, carbon of soil (%); N, nitrogen of soil (%); CN, C/N radio of soil; LWC, leaf water content.

**Fig.S3 The results of redundancy analysis between all species and environmental factors.** The numbers in the circle represent the interpretation rate of the factor for leaf water content. MAT, mean annual temperature; MAP, mean annual precipitation; C/N, C/N radio of soil.
